# Spatiotemporal and Quantitative Monitoring of the Fate of ‘*Candidatus* Phytoplasma Solani’ in Tomato Plants Infected by Grafting

**DOI:** 10.3390/pathogens10070811

**Published:** 2021-06-26

**Authors:** Gaia Carminati, Vittorio Brusa, Alberto Loschi, Paolo Ermacora, Marta Martini

**Affiliations:** Department of Agricultural, Food, Environmental and Animal Sciences (DI4A), University of Udine, Via delle Scienze 206, 33100 Udine, Italy; carminati.gaia@spes.uniud.it (G.C.); brusa.vittorio@gmail.com (V.B.); alberto.loschi@uniud.it (A.L.); paolo.ermacora@uniud.it (P.E.)

**Keywords:** stolbur, multiplication dynamic, distribution, tomato

## Abstract

Understanding how phytoplasmas move and multiply within the host plant is fundamental for plant–pathogen interaction studies. In recent years, the tomato has been used as a model plant to study this type of interaction. In the present work, we investigated the distribution and multiplication dynamics of one strain of ‘*Candidatus* Phytoplasma (*Ca*. P.) solani’ (16SrXII-A) in tomato (*Solanum lycopersicum* L., cv. Micro-Tom) plants. We obtained infected plants by grafting, a fast and effective method to maintain phytoplasma infection. *In planta* spread and multiplication of ‘*Ca*. P. solani’ was monitored over time using qualitative and quantitative qPCR. Root, apical shoot, lower leaves, and upper leaves were sampled at each sampling time. We hypothesized that ‘*Ca*. P. solani’ from the grafting site reached firstly the highest leaf, the apex and the roots; subsequently, the phytoplasmas spread to the rest of the upper leaves and then progressively to the lower leaves. Significant differences were found in ‘*Ca*. P. solani’ titer among different plant tissues. In particular, the concentration of phytoplasma in the roots was significantly higher than that in the other plant compartments in almost all the sampling dates. Since the roots show rapid colonization and the highest concentration of phytoplasmas, they represent the ideal tissue to sample for an early, sensitive and robust diagnosis.

## 1. Introduction

Phytoplasmas are phloem-limited bacteria, belonging to the class of *Mollicutes*. In the natural environment, they are transmitted by phloem-feeding insects (Hemipters) thanks to their trans-kingdom ability to invade and multiply in both plant and animal cells [1]. Phytoplasmas are associated with diseases causing damages in hundreds of plant species, including economically relevant crops and fruit tree cultures [2,3].

‘*Candidatus* Phytoplasma (*Ca*. P.) solani’ is associated in Europe and in the Mediterranean basin with bois noir (BN) disease in grapevine and with stolbur (STOL) diseases in wild and cultivated herbaceous and woody plants. It has been formally described within the 16SrXII group [4] together with other four species: (i) ‘*Ca*. P. australiense’ [5]; (ii) ‘*Ca*. P. japonicum’ [6]; (iii) ‘*Ca*. P. fragariae’ [7] and (iv) ‘*Ca*. P. convolvuli’ [8].

‘*Ca*. P. solani’ can be transmitted both by vegetative propagation of infected hosts and by sap feeding insect vectors of the families Cixiidae [9,10] and Cicadellidae [11], whose most common vector is represented by *Hyalesthes obsoletus*, a polyphagous planthopper. In experimental conditions, however, grafting represents the faster and most effective method to maintain ‘*Ca*. P. solani’ infection in experimental plants.

The tomato plant, besides being an economically important crop, is a natural plant host of several phytoplasmas worldwide [12,13]; one of the most important in Europe is ‘*Ca*. P. solani’ [14]. The sequencing of its genome in 2012 [15] has improved its use as a model plant and in recent years, it has been used as such also for studies on ‘*Ca*. P. solani’–plant interaction [16,17,18,19,20]. Among the different tomato cultivars, cv. Micro-Tom is particularly indicated for scientific research due to its small size and rapid growth.

Understanding how phytoplasmas move and multiply within the host plant is fundamental for plant–pathogen interaction studies [21,22]. In the last 20 years, PCR and quantitative PCR have been used to reveal how phytoplasmas move, distribute and multiplicate in plants and how they are related to symptom expression in several host plants, including herbaceous species, infected by phylogenetically different phytoplasmas [23,24,25,26,27,28,29].

In the present work, we investigated the distribution and multiplication dynamics of one strain of ‘*Ca*. P. solani’ (16SrXII-A) in tomato plants (*Solanum lycopersicum* L., cv. Micro-Tom) infected by grafting. The fate of ‘*Ca*. P. solani’ has been monitored over time using a relative quantification method (‘*Ca*. P. solani’ genome units per nanogram of plant DNA) based on real-time PCR (qPCR) assay [28].

## 2. Results

### 2.1. ‘Ca. P. solani’ Detection

**Figure 1:**
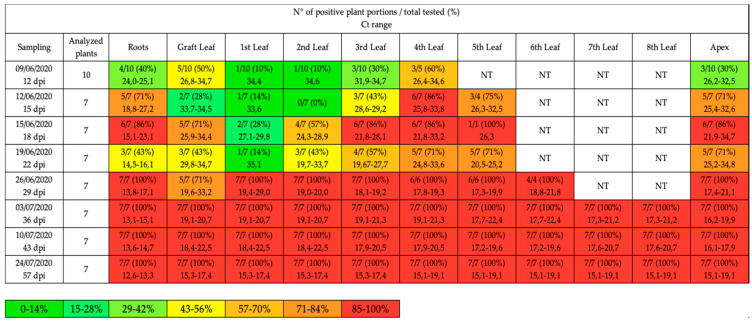
Qualitative qPCR results obtained in each sampling date and expressed as the number of positive plant portions on the total number of tested portions and relative percentage (%). The leaves are numbered in a bottom-up manner, the 1st leaf represents the first leaf found after the grafting site. The values of C_t_ are reported as range considering positive samples with C_t_ < 35 cycles. The colors indicate the different percentages; NT = not tested because not available due to the plant development.

In the present study, a total of 59 plants were analyzed. The first sampling (12 dpi) allowed to confirm the beginning of the infection (Figure 1), though it was not included in the quantitative analysis. The first signs of suspicious symptoms (yellowing and erect bearing of the inflorescences) were registered around 15 June (18 dpi), but the development of pathognomonic symptoms (virescence, cauliflower-like inflorescences) was recorded only on 21 June (24 dpi). At 29 dpi, the presence of big bud symptoms was registered and after 36 dpi the growth pattern began to show signs of disruption, with evident loss of the apical dominance and lateral shoot proliferation.

**Figure 2:**
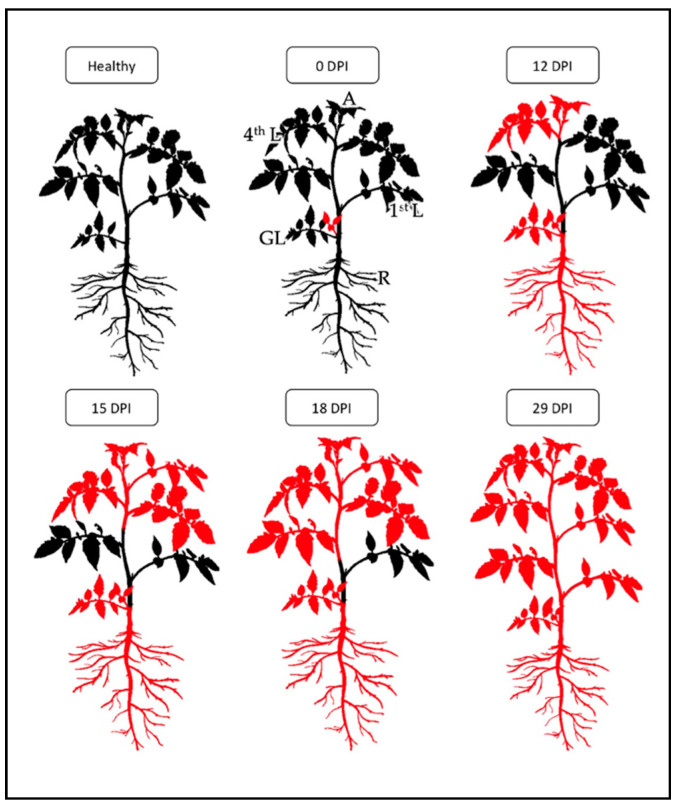
Reconstruction of *in planta* hypothetical migration of ‘*Ca*. P. solani’ starting from inoculation by grafting (0 dpi) until systemic infection (29 dpi). The portions colored in red indicate the localization of ‘*Ca*. P. solani’ within the plant. Healthy: healthy plant; 0 dpi: lateral grafting; 12 dpi: colonization of 4th leaf (L), root (R) and apex (A); 15 dpi: colonization of 3rd L; 18 dpi: colonization of 2nd L; 29 dpi: ‘*Ca*. P. solani’ present in all parts of the plant.

The results of the qualitative qPCR are summarized in Figure 1 and Figure 2; all samples with C_t_ value <35 and melting temperature (Tm°) between 79.4 °C and 79.8 °C were considered positive (data not shown). At 12 dpi, different portions of tomato plants had been colonized by ‘*Ca*. P. solani’, in particular the leaf closest to the graft (GL) and the highest leaf (4th L), with an onset of infection in the roots (R) and in the apexes (A) of some plants.

At 15 dpi, almost all of the apexes and roots had been colonized, as well as the higher leaves (4th L and 5th L). At 18 dpi and 22 dpi, the infection continued towards the center of the plant, reaching the central leaves (3rd L and 2nd L) in most plants.

At 29 dpi, the characteristic symptoms were now present on most of the plants, allowing to abandon the completely randomized method of the first samplings and to test only symptomatic plants: from this point on, all the plants chosen resulted positive in each portion (systemic infection).

Figure 2 shows the schematic representation of the hypothetical migration of ‘*Ca*. P. solani’ in tomato starting from inoculation by grafting (0 dpi) until systemic infection is reached (29 dpi).

The identification of positive samples allowed us to determine which plants to process with quantitative qPCR especially in the early stages of infection when symptoms were not yet well developed. Moreover, we also decided to separate the DNA samples of each plant into 4 compartments (apex, roots, upper leaves and lower leaves), thus some diluted DNAs obtained from the leaves were joined together to form the DNA pools of the lower and upper parts, while the root and apex DNAs were kept unaltered.

### 2.2. ‘Ca. P. solani’ Quantification

With qPCR analysis it was possible to evaluate the ‘*Ca*. P. solani’ concentration and its multiplication dynamics in 4 different plant compartments. To make the C_t_ values of the different plates comparable, the values of the 4 samples used as replicate calibrators in each plate were evaluated. The mean variation coefficient (vc) of the C_t_ values was found to be 0.49 cycles for the LL_C_ (lower leaves calibrator), 0.71 cycles for UL_C_ (upper leaves calibrator), 0.24 cycles for R_C_ (roots calibrator) and 1.37 cycles for A_C_ (apex calibrator). These values denote a high degree of reproducibility of the PCR reactions, which allowed the comparison of the C_t_ values obtained within different qPCR runs.

For the quantification of ‘*Ca*. P. solani’ DNA (expressed in genomic units, GU), a conversion of the plasmid DNA concentration from ng/μL to the number of genomic units was performed.

The quantification of ‘*Ca*. P. solani’ in infected samples, was obtained by extrapolating from the standard curve the number of genomic units (GU) and normalizing these data with the total genomic DNA expressed in ng and determined by the standard curve.

The results of the quantification are summarized in Table 1 and the complete data obtained for each sample from the 4 different compartments were reported respectively in Table S1 of the Supplementary Materials.

**Figure 3:**
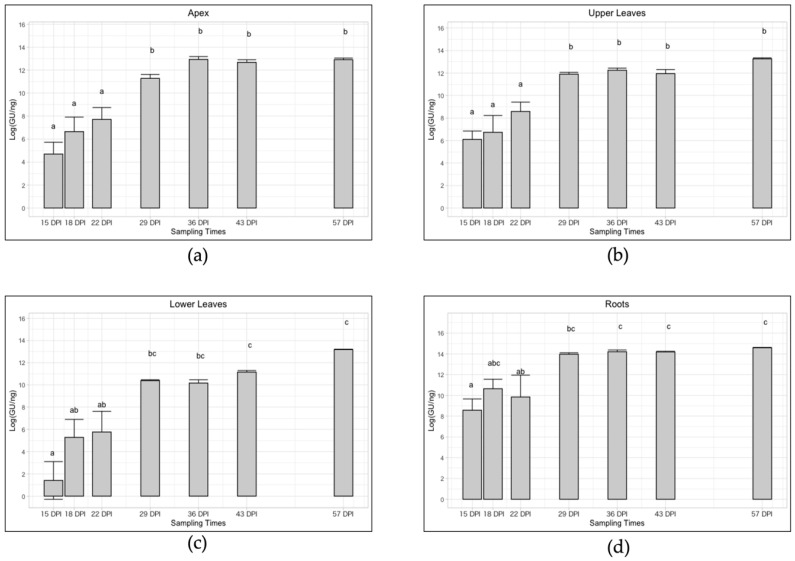
Trend of ‘*Ca*. P. solani’ infection (expressed as Log of ‘*Ca*. P. solani’ GU/ng of genomic DNA) at different sampling times in four different plant compartments: apex (**a**), upper leaves (**b**), lower leaves (**c**), roots (**d**). Different letters above bars (a, b, c, ab, bc, abc) indicate statistically significant differences according to the Siegel–Tukey post hoc test, *p* < 0.05. Error bars indicate Standard Error of the Mean of 5 biological replicates for each sampling date.

As shown in Figure 3 the trend of infection for the four plant-compartments followed the same pattern with decreasing C_t_ values which underlined an increase in the phytoplasma titer. In particular, the apexes showed an average relative concentration from 6.95 × 10^2^ to 4.19 × 10^5^ ‘*Ca*. P. solani’ GU/ng genomic DNA, with a significant increase of exactly 602 times (Table 1 and Figure 3). In the upper part, the average value increased significantly in a similar way, precisely 417 times, from 1.40 × 10^3^ to 5.84 × 10^5^ ‘*Ca*. P. solani’ GU/ng genomic DNA (Table 1 and Figure 3), while in the lower part it increased significantly by almost 7000 times (precisely 6957), from 7 × 10^1^ to 4.87 × 10^5^ ‘*Ca*. P. solani’ GU/ng genomic DNA (Table 1 and Figure 3). In the roots tissue a significant concentration increase of 140 times was observed over the time span of the sampling, from an initial concentration of 1.55 × 10^4^ ‘*Ca*. P. solani’ GU/ng genomic DNA up to 2.18 × 10^6^ in the last sampling (Table 1 and Figure 3).

**Figure 4:**
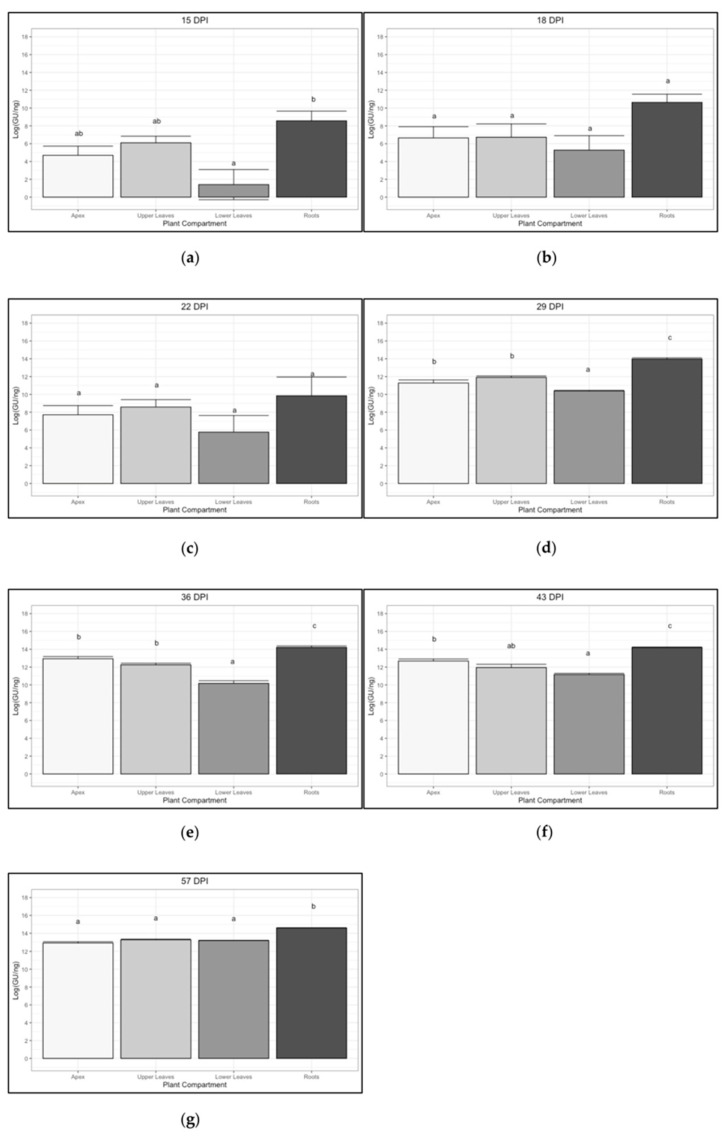
Trend of ‘*Ca*. P. solani’ infection (expressed as Log of ‘*Ca*. P. solani’ GU/ng of genomic DNA) in four different plant compartments at different sampling times: 15 dpi (**a**), 18 dpi (**b**), 22 dpi (**c**), 29 dpi (**d**), 36 dpi (**e**), 43 dpi (**f**) and 57 dpi (**g**). Different letters above bars (a, b, c, ab) indicate statistically significant differences according to the Siegel–Tukey post hoc test, *p* < 0.05. Error bars indicate Standard Error of the Mean of 5 biological replicates for each compartment.

As shown in Figure 4, the concentration of phytoplasma in the roots was significantly higher than that in the other plant compartments in all the sampling dates with the exception of those at 18 and 22 dpi. In particular, the initial average concentration (15 dpi) of the roots was 22 times that of the apexes, 11 times that of the upper part, and 221 times higher than that of the lower part. At the last sampling (57 dpi) the ‘*Ca*. P. solani’ concentration reached similar values (4.19 × 10^5^–5.84 × 10^5^) in all the other compartments different from the roots which still remained 3.73–5.2 times more concentrated.

## 3. Discussion

This work aimed to describe how ‘*Ca*. P. solani’ moves and multiplies inside the host plant *S. lycopersicum* cv. Micro-Tom after controlled inoculation by grafting.

The use of the test plant *S. lycopersicum* cv. Micro-Tom was confirmed as an excellent choice because it is easy to maintain under controlled conditions, it grows quickly occupying small spaces and it shows specific symptoms that occur about a month after infection.

In this type of study, it was essential to keep the infection conditions as controlled as possible: localized grafting with material from symptomatic plants that were homogeneous in terms of age and time of infection, the same time of inoculation and the same age of the test plants in all experiments. Due to the fact that phytoplasmas cannot be cultivated, mechanical inoculation can only be carried out by grafting, or experimental vector insects can be used to reproduce the infection conditions most similar to natural ones.

Scientific literature tends to favor localized transmission via vector insect, as it is closer to the diffusion dynamics present in nature. However, in our case, we were not aware of an experimental method developed for the infection of tomato with ‘*Ca*. P. solani’ through an insect vector, therefore our experimental work was based on the transmission of ‘*Ca*. P. solani’ by grafting.

Among the advantages of this technique, we should mention the relative simplicity of execution, the high percentage of transmission, as well as the reduced requirements in terms of space and of special equipment compared to those necessary for transmission by the insect vector.

The results obtained from the qualitative analysis allowed us to hypothesize the following diffusion model of ‘*Ca*. P. solani’ in tomato: from the graft, the phytoplasmas have migrated into the main stem to reach the highest leaf, the apex and the roots which were the first portions to be colonized (12 dpi). Subsequently, the phytoplasmas spread to the rest of the young leaves of the upper part (15 dpi) and then from the top of the plant progressively to the old leaves of the lower part (22 dpi and 29 dpi). After 29 dpi the plants were infected in all portions, so we can conclude that ‘*Ca*. P. solani’ in the tomato takes about 4 weeks to become systemic. This distribution dynamic is consistent with previous findings on *Chrysanthemum coronarium* [25,26], where an initial apex and root infection and the subsequent colonization of phytoplasmas from top to bottom of the plant were described.

The proposed model for ‘*Ca*. P. solani’ colonization considered both the number of samples per plant portion resulted positive and their relative cycle threshold (Ct), thus a custom made index was developed to consider both variables. The threshold index was set as 1.0 with a PP value equal to 35% and a $\bar{C_{t}}$ value of 35 cycles with the aim to describe ‘*Ca*. P. solani’ distribution also at the early stages of infection. As shown by the supplementary Figure S1, the pattern of colonization can be approximately reconstructed as in Figure 2.

After infection through grafting, it is difficult to study the phytoplasma movement and multiplication during the first stages of infection when the symptoms are not yet visible, due to the fact that even using evenly infected material, the graft might weld at different times. Thus, at the early stages of infection, the colonization rates between infected plants could be slightly different; this is the reason why the quantification data of the initial samplings (until 26 June 2020, 29 dpi) showed a higher dispersion. However, the 15 dpi-analysis clearly showed that the highest phytoplasma titer is contained in the roots, followed by the apex and the upper leaves compartments; at this stage, all three compartments are composed of sink tissues, whether the sink function is due to their age (apex and upper leaves) or their role (roots). Thus, it seems that during the first stages of colonization the movement of the phytoplasmas was limited to sink units; only after colonizing these portions did the infection spread systemically through the whole plant.

Subsequently, the phytoplasma increased its concentration in all the plant compartments following a similar trend (Figure 3). In particular, in the roots that have always been very concentrated, the increase was smaller (140 times), while in the lower part, it went from an extremely low initial concentration to a concentration 7000 times higher and equal to that of the apexes and of the upper part colonized previously. The latter tissues showed a similar increase in concentration of 602 and 417 times, respectively.

Analyzing all the sampling dates (Figure 4), the ‘*Ca*. P. solani’ titer is often shown to be significantly higher in the root compartment over all the others. Exceptions are represented by the 18-dpi and 22-dpi samplings, although the wide dispersion of the data before the development of pathognomonic symptoms does not allow a fine distinction between significance classes. The lower leaves compartment, which at the beginning only included leaves that function as sources, was shown to be the least invaded tissue in the early phases of colonization. However, at the last stages of colonization (57 dpi), the ‘*Ca*. P. solani’ titer equaled that of the other compartments excluding the roots.

These findings indicate that especially in the roots, but also in the apexes and upper leaves, phytoplasmas are actively multiplying in agreement with [25,26,30]. This would lead us to hypothesize that from the inoculation site the phytoplasmas quickly reach the roots and apexes, where the intense metabolic activity and the high availability of photosynthates allow them to multiply very quickly and then spread over the rest of the plant. This dynamic is compatible with the model according to which the movement of phytoplasmas is a process largely explained by the mass flow in the phloem [21,31,32]. Nevertheless, the movement of phytoplasmas cannot be solely explained by mass flow as suggested in van Bel and Musetti [32].

The very fast colonization of the roots and the apexes makes them ideal to sample for early detection of phytoplasmas, when the symptoms have not yet developed. Moreover, the roots presented the highest concentration of phytoplasmas throughout the entire sampling period, therefore a diagnostic method based on root sampling would be quite sensitive and robust. Although, when working with root tissue, it has to be kept in mind that the nucleic acid extracts may have some inhibition issues linked to the presence of humic acid residues or other inhibitors [25].

This colonization and multiplication pattern of ‘*Ca*. P. solani’ in tomato can be very useful not only in suggesting the best time and tissue for a sensitive and accurate diagnosis, but also to understand the relationships between plant and pathogen especially in this case, in which the model plant is also a natural host for the pathogen. Such an experimental model in our research group could be applied to study the fate of different strains of phytoplasma in single or mixed infection in the same host plant or the fate of the same phytoplasma strain in different *Solanaceae* species.

## 4. Materials and Methods

### 4.1. Phytoplasma Strain, Plant Material and Grafting

A strain of ‘*Candidatus* Phytoplasma (*Ca*. P.) solani’(*tuf* genotype tuf-b) maintained in tomato (cv. Micro-Tom) was used for the study. The strain was provided by the Institute for Sustainable Plant Protection (IPSP), Turin, and originally found in tomatoes in Puglia (South Italy). The plant material for the trial was obtained by inoculation via homologous grafting. A total of 96 40-day old (4-true-leaf stage) plants of tomato (cv. Micro-Tom) were inoculated with the ‘*Ca*. P. solani’ strain maintained in the same cultivar. Grafting was performed on 28th May 2020 in the stem at the level of the first true-leaf with symptomatic shoots derived from 13 infected plants. The source plants had similar conditions of ‘*Ca*. P solani’ infection showing a maximum difference of C_t_ equal to 1.6 cycles. All the grafted plants were kept under controlled conditions in an experimental greenhouse at 24 ± 3 °C and with long photoperiod conditions with 14–16 h of light [33].

### 4.2. Sampling Scheme

Plants were sampled at irregular intervals and precisely at: 12 (9 June 2020); 15 (12 June 2020); 18 (15 June 2020); 22 (19 June 2020); 29 (26 June 2020); 36 (3 July 2020); 43 (10 July 2020) and 57 (24 July 2020) dpi (days post-infection). The first sampling (performed once the graft was welded) was used to confirm the beginning of the infection and was not included in the quantitative analysis. The three following dates of sampling aimed to describe in detail the ‘*Ca*. P. solani’ distribution during the first stages of infection. Three more samplings were used to estimate the concentration growth curve at 7-days intervals, while the last one was used to confirm that the ‘*Ca*. P. solani’ titer had reached the plateau-phase. All samplings were planned to randomly choose 7 plants each time with the sole exception of the first sampling (10 plants). To allow the comparison between different plants and especially between those sampled before the onset of symptoms, a surplus of plants was sampled and only those plants showing positivity (Ct < 35) in more than half of their tissues were kept for the quantitative analysis. When most of the plants began to show characteristic symptoms of the disease (around 21 June, 24 dpi), about a dozen plants were discarded because they had no reliable symptoms and/or because their development was rather stunted. So from that moment on, the sampling was done only on symptomatic plants. Each plant was subdivided into a variable number of subsamples that comprehended: (i) one sample for the apex portion; (ii) one sample for the root portion; (iii) a number of samples equal to the number of leaves of the plant (one for each).

### 4.3. Nucleic Acid Extraction and ‘Ca. P. solani’ Detection

All the subsamples, except the radical ones, were prepared to enrich the phloem tissue portion, weighed and stored at −20 °C. Total DNAs were extracted from each subsample using a CTAB extraction method modified [34] from Doyle and Doyle [35]. From 0.5 g to 1 g of frozen tissue per sample was used. Quality and quantity of extracted DNA were assessed by means of NanoDrop 1000 Spectrophotometer (Thermo Scientific, Wilmington, DE, USA) and each subsample concentration was adjusted to 20 ng/μL by dilution in nuclease-free water. In each subsample, the presence of ‘*Ca*. P. solani’ was accessed by qualitative qPCR and the identity of the strain was confirmed by high-resolution melt (HRM) analysis. Qualitative qPCR was performed using 16S rDNA as a target by means of phytoplasma universal primers 16S(RT)F1/16S(RT)R1 (5′-TTC GGC AAT GGA GGA AAC T-3′)/(5′-GTT AGC CGG GGC TTA TTC AT-3′) (fragment 138 bp long) [36]. qPCRs were performed in a final reaction volume of 15 μL per reaction in a 96-well Bio-Rad CFX96 RealTime PCR System (Bio-Rad Inc., Hercules, CA, USA), in white-walled PCR plates with clear adhesive sealers.

Reaction mixtures contained 0.3 μM each primer, 1X SsoFast™ EvaGreen ^®^ Supermix (Bio-Rad Inc., Hercules, CA, USA), molecular grade H_2_O; 2 µL of DNA solution containing 20 ng/μL of extracted DNA as a template. Cycling conditions were as follows: initial denaturation at 98 °C for 2 min; 55 cycles of 5 sec at 98 °C; 5 sec at 57 °C. A high resolution melting curve analysis (ramp from 65 °C to 95 °C with 0.2 °C temperature increments and holding time of 10 sec) was programmed at the end of the cycling reaction to evaluate the purity of the amplification product.

To propose a model for ‘*Ca*. P. solani’ colonization we decided to consider both the number of samples per plant portion that resulted positive and their relative cycle threshold (C_t_). A custom made index was developed to consider both variables:

$$i\;\propto \frac{PP}{\bar{C_{t}}}$$

where *PP* is the percentage of the positive samples and $\bar{C_{t}}$ is the mean cycle threshold of the positive samples. Since all the analyses were destructive, the resulting index was evaluated not just by itself, but also in comparison with previous and next sampling. The threshold index was set as 1.0 with a PP value equal to 35% and a mean C_t_ value of 35 with the aim to describe ‘*Ca*. P. solani’ distribution also at the early stages of infection. A summarization of the indicator with its value and related heatmap is provided in Table S2 of the Supplementary Materials.

### 4.4. ‘Ca. P. solani’ Quantification

For the quantitative analysis, five plants out of the seven tested with qPCR were chosen. For the first sampling dates, the plants with more than half of the plant portions that resulted positive were preferred, whereas for the following dates when infection became nearly or completely systemic the plant choice was randomized. If necessary, the subsamples of the chosen plants were pooled in equal volumes to represent the following 4 compartments: the apex (no pooling), the upper leaves (high leaves pooling), the lower leaves (bottom leaves pooling) and the roots (no pooling). The titer of ‘*Ca*. P. solani’ in the 4 plant compartments was determined by qPCR as number of ‘*Ca*. P. solani’ genome units (GU)/ng of plant DNA to normalize the data [28].

As for the qualitative analysis, 16S rDNA was used as a target for qPCR of ‘*Ca*. P. solani’; whereas the 18S rDNA was chosen as a target for the qPCR of plant DNA [29]. ‘*Ca*. P. solani’ and plant DNAs were amplified with primer pairs 16S(RT)F1/16S(RT)R1 [36] and Plant 18S f/r (5′-GACTACGTCCCTGCCCTTTG3′)/(5′-AACACTTCACCGGACCATTCA-3′) [29], respectively.

To quantify ‘*Ca*. P. solani’ DNA, a standard curve was established by diluting a plasmid pGEM^®^ Easy Vector (Promega, Madison, WI, USA) containing the 16S rRNA gene of Flavescence dorée (FD) phytoplasma (quantified by using Qubit^®^ 2.0 Fluorimeter). Serial dilutions (1:10) of the plasmid starting from 1 ng/μL to 1 fg/μL were prepared in sterile H_2_O. PCRs mixtures and cycling conditions were performed as described above.

Similarly, to quantify plant DNA, a standard curve was prepared with 10-fold serial dilutions of total DNA from a healthy plant (quantified by using Qubit^®^ 2.0 Fluorimeter), starting at 5 ng/μL and up to 50 fg/μL. PCRs mixtures were prepared as described before. Cycling conditions for primers Plant 18S f/r were as follows: initial denaturation at 98 °C for 2 min; 45 cycles of 5 s at 98 °C; 5 s at 64 °C. A low-resolution melting curve (ramp from 65 °C to 95 °C with 0.5 °C increments and holding times of 5 s) was programmed at the end of the cycling reaction.

The amount of fluorescence for each sample was measured at the end of each cycle and analyzed via CFX-Manager Software v. 2.0 (Bio-Rad Laboratories, Inc., Hercules, CA, USA). The baseline was automatically determined and the fluorescence threshold was set manually to maximize the standard curve efficiency. Each diluted sample and each standard were replicated twice in the experiment. In each plate were also inserted a negative control, free of phytoplasma DNA, and 4 calibrators, consisting of a positive sample for each type of tissue (A_C_ = apex calibrator, LL_C_ = lower leaves calibrator, UL_C_ = upper leaves calibrator and R_C_ = root calibrator), whose analysis was repeated in each plate to verify the reproducibility of the results, which only in this way could they be compared between the four qPCRs. The variation coefficient (vc) between the values of the calibrators in the different plates was calculated as follows [37]:

$$vc=\frac{\sigma }{\mu }\cdot 100$$

where *vc* is the variation coefficient; *σ* is the standard deviation of the C_t_ values of a calibrator in different plates; and *μ* represents the mean of the C_t_ values of the same calibrator in different plates.

## 5. Data Analysis

The ‘*Ca*. P. solani’ concentration was expressed as the ratio of ‘*Ca*. P. solani’ genome units (GU) present in each sample versus the nanograms (ng) of the plant DNA of the same sample to normalize the data. Observational errors were calculated as the higher value between the maximum semi-dispersion and the instrument sensibility for the technical replicas (Supplementary Table S1) of ‘*Ca*. P. solani’ GU and plant DNA and as the standard error between the five biological replicas for each compartment (Table 1, Figure 3 and Figure 4). For the ratio between ‘*Ca*. P. solani’ GU and plant DNA, the uncertainty propagation was estimated as the maximum semi-dispersion between the maximum and the minimum values derived by the ratio.

To compare the infection rates at different times and among different plant compartments, analysis of variance (ANOVA) was performed on normalized data.

Normalization was performed by means of a box cox transformation on the whole set of data and normality was then tested (Shapiro–Wilk normality test) on each data group, defined as the total of samples afferent to the same date and plant compartment. Homoskedasticity was verified by Levene Test for each variable (date and plant compartment). For visualization, data were reported as the logarithm of the ratio (calculated as previously described) and the error bar was calculated as standard deviation. After performing the analysis of variance, a comparison of means was determined by Tukey’s test (*p* ≤ 0.05). All the statistical analyses were performed by means of R studio software, v4.0.2 [38].

## Figures and Tables

**Table 1:** Data of average relative concentration expressed as ‘*Ca*. P. solani’ genome units (GU)/ng of genomic DNA ± Standard Error (SE) in four plant compartments at different sampling dates.

Date	Apex	Upper Leaves	Lower Leaves	Roots
	GU/ng Genomic DNA ± SE			
12 June 2020 15 dpi	6.95 × 10^2^ ± 5.90 × 10^2^	1.40 × 10^3^ ± 1.08 × 10^3^	7.00 × 10^1^ ± 4.23 × 10^1^	1.55 × 10^4^ ± 6.43 × 10^3^
15 June 2020 18 dpi	2.89 × 10^3^ ± 1.36 × 10^3^	1.17 × 10^4^ ± 9.14 × 10^3^	2.12 × 10^3^ ± 1.19 × 10^3^	1.12 × 10^5^ ± 5.34 × 10^4^
19 June 2020 22 dpi	6.71 × 10^3^ ± 3.52 × 10^3^	1.58 × 10^4^ ± 1.02 × 10^4^	3.44 × 10^3^ ± 2.61 × 10^3^	3.93 × 10^5^ ± 1.95 × 10^5^
26 June 2020 29 dpi	1.01 × 10^5^ ± 3.63 × 10^4^	1.55 × 10^5^ ± 2.43 × 10^4^	3.29 × 10^4^ ± 2.53 × 10^3^	1.21 × 10^6^ ± 1.58 × 10^5^
3 July 2020 36 dpi	4.75 × 10^5^ ± 1.42 × 10^5^	2.23 × 10^5^ ± 3.86 × 10^4^	3.12 × 10^4^ ± 9.63 × 10^3^	1.58 × 10^6^ ± 3.14 × 10^5^
10 July 2020 43 dpi	3.48 × 10^5^ ± 5.83 × 10^4^	2.02 × 10^5^ ± 7.49 × 10^4^	7.31 × 10^4^ ± 1.17 × 10^4^	1.47 × 10^6^ ± 8.55 × 10^4^
24 July 2020 57 dpi	4.19 × 10^5^ ± 5.61 × 10^4^	5.84 × 10^5^ ± 5.00 × 10^4^	4.87 × 10^5^ ± 4.57 × 10^4^	2.18 × 10^6^ ± 6.18 × 10^4^

## Data Availability

Not applicable.

## Supplementary Materials

The following are available online at https://www.mdpi.com/article/10.3390/pathogens10070811/s1, Table S1: extended report of the quantitative analyses to determine concentration of ‘*Ca*. P. solani’ in tomato, Table S2: values of the custom made indicator (i) used to evaluate the level of ‘Ca. P. solani’ infection in tomato.
